# Supplementary figures and images for: Elevated liver glycogenolysis mediates higher blood glucose during acute exercise in Barth syndrome
Source: PLoS One. 2023 Aug 31;18(8):e0290832. doi: 10.1371/journal.pone.0290832 (PMC10470866; doi:10.1371/journal.pone.0290832)

## Slide 1
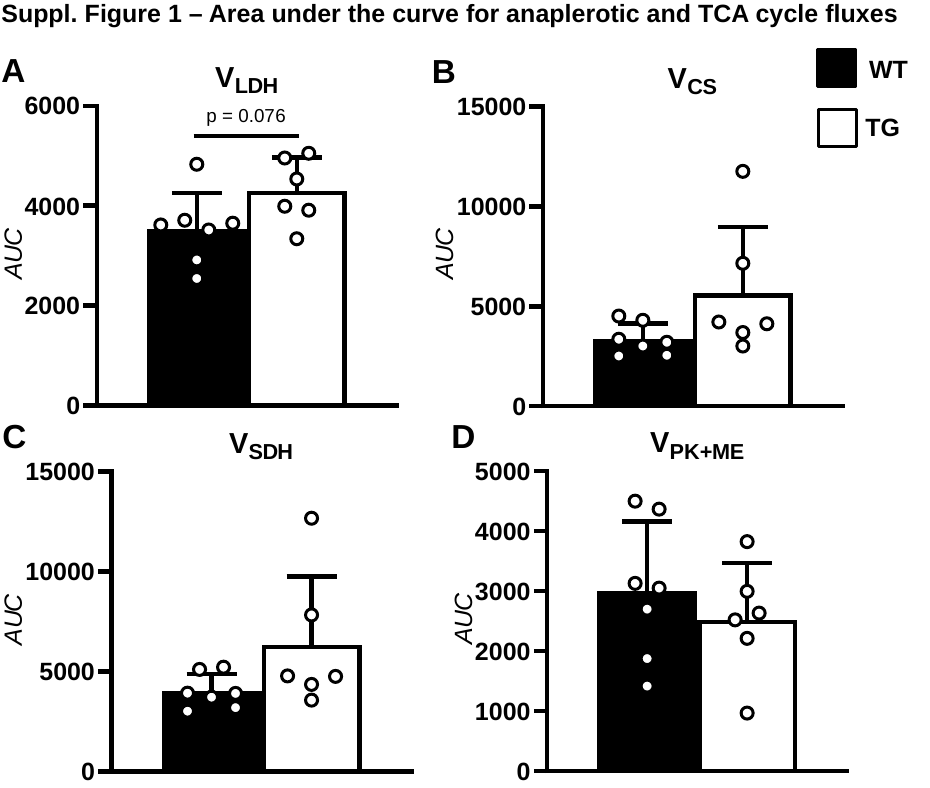

Suppl. Figure 1 – Area under the curve for anaplerotic and TCA cycle fluxes
A
B
WT
TG
C
D

Supplement: S1 Fig — (PPTX) [file pone.0290832.s001.pptx]
